# AIDM-Strat: Augmented Illegal Dumping Monitoring Strategy through Deep Neural Network-Based Spatial Separation Attention of Garbage

**DOI:** 10.3390/s22228819

**Published:** 2022-11-15

**Authors:** Yeji Kim, Jeongho Cho

**Affiliations:** Department of Electrical Engineering, Soonchunhyang University, Asan 31538, Republic of Korea

**Keywords:** waste disposal, object detection, multi-object tracking, articular point, garbage bag

## Abstract

Economic and social progress in the Republic of Korea resulted in an increased standard of living, which subsequently produced more waste. The Korean government implemented a volume-based trash disposal system that may modify waste disposal characteristics to handle vast volumes of waste efficiently. However, the inconvenience of having to purchase standard garbage bags on one’s own led to passive participation by citizens and instances of illegally dumping waste in non-standard plastic bags. As a result, there is a need for the development of automatic detection and reporting of illegal acts of garbage dumping. To achieve this, we suggest a system for tracking unlawful rubbish disposal that is based on deep neural networks. The proposed monitoring approach obtains the articulation points (joints) of a dumper through OpenPose and identifies the type of garbage bag through the object detection model, You Only Look Once (YOLO), to determine the distance of the dumper’s wrist to the garbage bag and decide whether it is illegal dumping. Additionally, we introduced a method of tracking the IDs issued to the waste bags using the multi-object tracking (MOT) model to reduce the false detection of illegal dumping. To evaluate the efficacy of the proposed illegal dumping monitoring system, we compared it with the other systems based on behavior recognition. As a result, it was validated that the suggested approach had a higher degree of accuracy and a lower percentage of false alarms, making it useful for a variety of upcoming applications.

## 1. Introduction

Economic and social progress in the Republic of Korea resulted in an enhanced standard of living, which subsequently led to enormous amounts of waste from enriching consumer goods. A significant societal issue is created by this rise in garbage levels, which also harms the environment [1]. Additionally, used-up household items, garbage, and construction waste produce foul odors and pollutants, ruining the urban landscape and threatening citizens’ health. To address this issue and develop a clean, garbage-less environment, the government implemented a volume-rate waste disposal system in 1995. The new program has a pricing model that enables people to bear a volume-rate cost from their garbage to voluntarily reduce waste and maximize the separate disposal of recyclable items, in contrast to the existing program that imposed incremental fees based on the sizes of houses or the rate of property tax [2].

Waste eligible for volume-rate disposal corresponds to municipal waste generated by households and small enterprises. Standardized volume-based bags must be purchased to dispose of waste. As a motivation for minimizing a pollutant’s effect on health and the environment and an economic incentive to improve optimal waste disposal and increase knowledge of the citizens, the volume-based garbage disposal system aims to convey a need for the reduction of illegal garbage dumping and the cooperation and participation [3]. The method can lessen the burden and cost associated with gathering, moving, and processing waste. However, regular instances of illegal rubbish dumping are caused by the bother of having to purchase conventional garbage bags on one’s own and the challenging process of handling enormous waste. The uncovered cases of illegal garbage dumping in Seoul went from 99,098 in 2014 to 128,144 in 2020, revealing a year-on-year increase, and it is one of the numerous social problems that must be overcome [4]. Notable in particular are the rising instances of unlawful rubbish disposal in non-standard bags, such as white disposable delivery plastic bags or black disposable plastic bags, as more take-out food deliveries take place. Such illicit dumping is steadily increasing in the absence of aggressive prosecution, necessitating different measures.

**Figure 1:**
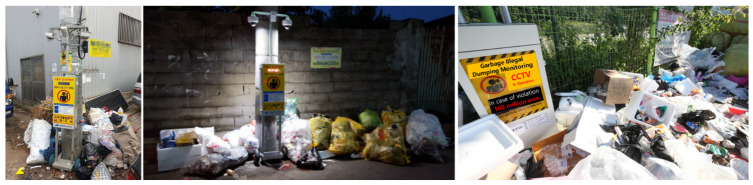
Limitations of the current illegal dumping monitoring system.

Watchpersons or government officials patrol to find illegal dumping situations occasionally, but such efforts need a larger labor force in wide areas. The recently installed closed-circuit television (CCTV) in locations with a concentration of unlawful dumping contains video recordings. However, the lack of manpower to conduct ongoing surveillance or analyze every single film makes it difficult to bring charges for illegal dumping [5]. Another comparable technique employs CCTV and human body identification sensors to send out an audio warning to onlookers to promote awareness, but the alert does not reveal illegal dumping; it causes noise disturbances due to the frequent pointless broadcasts. This approach may temporarily frighten illegal dumpers psychologically but has limited impacts in ending illegal dumping. Figure 1 depicts the illegal dumping monitoring system that is now in use with the CCTV and audio broadcasts as being surrounded by various forms of unlawfully placed rubbish. This demonstrates the limitations of the current illegal dumping monitoring system despite significant initial investment in the system.

Recently proposed methods combine deep-learning object detection technology widely in use with camera-based monitoring to monitor illegal dumping. The new approach can address the limitations of the existing methods requiring significant manpower and have the benefit of reducing unnecessary noise by enhancing false alarm rates. Min and Lee [6] proposed a way of catching illegal garbage dumping using a deep neural network trained on the joints of persons that are collected by image processing. By separating dumping postures from the other non-dumping postures, their system determines whether dumping is legal or illegal. Bae et al. [7] used the real-time object detection model, You Only Look Once (YOLO), to learn about the illegal dumping operation itself and to create zones for observation and non-observation in order to lower the system’s false alert rate. The trained model detects an act of dumping and then identifies it as illegal only when the coordinates of the activities are within the observation zone. Jeong et al. [8] used the Gaussian Mixture Model to examine object changes that are based on histogram differences. Their suggested approach is based on the idea that at the point of dumping, there is a divide between the dumper and the trash. Kim et al. [9] proposed a system that detects illegal dumping using probabilistic analysis of the object trajectory.

As a result, several techniques exist to track unlawful dumping using object detection and video analysis technologies based on convolutional neural networks (CNNs), as well as detecting sensors. Nevertheless, Refs. [6,7] consider an act of dumping as illegal when a non-dumping posture is similar to a dumping posture, even in the absence of garbage in hand, thus raising frequent false alarms. Therefore, Refs. [7,9] designated an observation zone for illegal dumping. As a result, their system cannot detect illegal dumping when it occurs outside of the surveillance zone and is susceptible to numerous missed detections. Therefore, Refs. [6,7,8,9] merely identifies characteristic changes of a dumper or only differentiates standard or non-standard garbage bags, which may raise a false alarm even when garbage is in a standard bag, all of which are issues still to be addressed. As a result, a more comprehensive monitoring system for unlawful dumping is required, one that goes beyond the dumping acts itself or isolated, small surveillance zones.

This study suggests a strategy of augmented illegal dumping monitoring (AIDM) that determines the distance between the dumper’s wrist and the garbage bag. To estimate the dumper’s wrist joint, Single Person Pose Estimation, which is a method for estimating spatial dependence combinations between body parts, is required and is largely divided into a tree-structured graphical model [10,11] and a non-tree model [12,13]. Afterward, CNN was applied to increase the reliability of joint estimation [14,15]. However, when two people are detected on one screen, the precise joint of each person cannot be extracted, so research on Multi-Person Pose Estimation [16,17] has been actively conducted. Among them, the OpenPose [18] model has been used in many fields and introduced in this study because it extracts joint points at a relatively high speed, and the amount of computation does not increase significantly even if the number of people increases.

The proposed method uses the OpenPose model [18] that can determine the articulation points of a person to extract the wrist joint and then uses the YOLO method [19] to classify four types of garbage bags. Additionally, to reduce errors from the unwarranted calculation of the distance of the wrist joint to the already dumped garbage bag or the issue of not identifying the same garbage due to the change in frames, we implement a Simple Online Realtime Tracking with A Deep Association Metric (DeepSORT) [20] that can keep track of multiple objects for tracking the garbage bag identifiers (IDs). We suggest an algorithm that can identify illegal dumping by keeping track of garbage bags that have already been dumped and those that are still to be dumped separately and deciding when the distance between the dumper’s wrist and the bag of trash is more than a certain threshold. The test findings demonstrate that our method of determining illegal dumping based on the distance of the actual dumper’s wrist to the garbage bag has better efficacy than other recently published methods that are based on behavior recognition or dumping zone designation. This research has the following contributions:

- With improved detection performance, the proposed monitoring system for illegal dumping can reduce noises caused by unnecessary audio guidance due to the inaccuracies of the existing illegal dumping broadcasting system;
- Using the object detection model, YOLO can differentiate the standard bags that are legal for garbage dumping and the other non-standard bags. Also, the proposed technique can minimize errors of falsely recognizing dumping-like behavior as illegal dumping through OpenPose, which can extract the articulation points;
- Our suggested method tracks the objects throughout the entire video without the use of specifically designated observation zones to evaluate whether illegal dumping happened;
- By introducing the object tracking model DeepSORT, we give IDs to already dumped garbage and garbage held in a dumper’s hand and track the objects to detect illegal dumping, thus lowering the missed detection rate.

In this Section, we discussed the need for an illegal dumping monitoring system and the goal of the study. In Section 2, we introduce the components of our illegal dumping monitoring system. In Section 3, we describe the design process of the proposed system. In Section 4, we describe the experimental conditions, testing, and results for the evaluation of the proposed system’s performance. In the last section, Section 5, we conclude our research.

## 2. Materials and Methods

**Figure 2:**
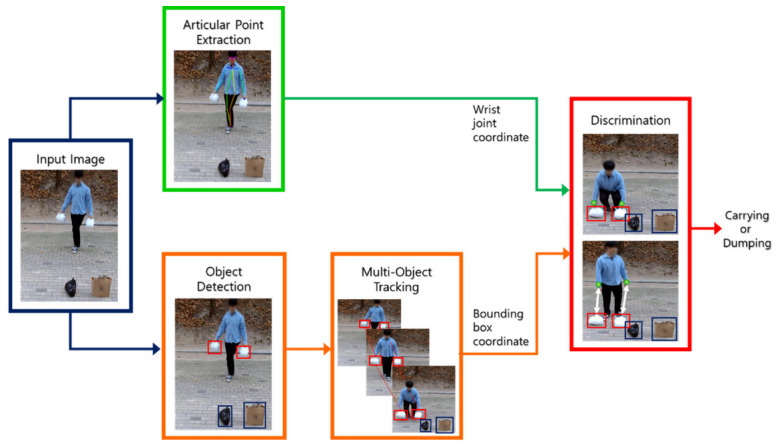
Schematic diagram of the proposed monitoring system for illegal dumping.

The monitoring system for illegal dumping that is presently in operation cannot decide on a dumping act itself for the illegality, and thus the impact is not as high as expected concerning the investment for the system implementation. Additionally, the systems that were recently designed using research on illicit dumping practices as the subject are highly susceptible to the probability of mistakenly associating suspicious behavior with unlawful dumping. As a result, we propose an improved monitoring system that identifies illegal dumping by classifying the types of garbage bags and estimating the distance of the dumper’s wrist to the garbage bag, as shown schematically in Figure 2. The object detector recognizes and classifies the rubbish bag while concurrently extracting a person’s joints from the input image. The object detector then begins tracking the garbage-classified object. Then, it continuously calculates the distance of the extracted wrist joint to the object detected as the non-standard bag. The dumping is considered unlawful if the distance exceeds a certain level.

### 2.1. Articular Point Extraction

Deep-learning-based posture estimation is done in two ways: the top-down technique for first finding an area with a person in it and then determining the posture in the area, and the bottom-up method for estimating the posture from the characteristic points of a human body without finding a person. To predict the joints, we employed the bottom-up OpenPose model in this study. Fast joint extraction is possible with OpenPose, and if more individuals are added, the calculation volume does not considerably rise, making it appropriate for crowded areas [21].

Based on a CNN, OpenPose infers characteristic points as joints and delineates them and uses VGGNet to enhance learning efficacy by extracting features of a wider area with fewer parameters. VGGNet creates a feature map F, which goes through a multilayered convolution branch α to create a confidence map γ representing the positions of the joints and goes through another branch β to create an affinity field δ indicating associations (location and direction) between body parts. The model first trains δ to obtain optimal δ predictions, which are then used to train γ. $\delta^{t}$ and $\gamma^{t}$ in the tth step are iterated by the respective branch $\beta^{t}$ up to the $T_{A}$ step, and then iterated by the branch $\alpha^{t}$ up to the $T_{A}+T_{B}$ Step, as summarized below [22]:

(1) $$\delta^{t}=\beta^{t}\left(F,\delta^{t-1}\right)\;,2\leq t\leq T_{A}$$

(2) $$\gamma^{t}=\alpha^{t}\left(F,\;\delta^{T_{A}},\;\gamma^{t-1}\right)\;,T_{A}<t\leq T_{A}+T_{B}$$

δ and γ obtained in each step are used to match a person’s arms and legs. The points finally determined as the person’s arms and legs are connected to extract the joints of the body. OpenPose learns through a loss function f composed of an objective function $f_{\delta }^{t}$ for the joint associations and another objective function $f_{\gamma }^{t}$ for the locations of the articular points. The object functions are as follows:

(3) $$f_{\delta }^{t}=\sum_{v=1}^{V}\sum_{P}W\left(P\right)\|\delta_{v}^{t}\left(P\right)-\delta_{v}^{*}{\left(P\right)\|}_{2}^{2}$$

(4) $$f_{\gamma }^{t}=\sum_{r=1}^{R}\sum_{P}W\left(P\right)\|\gamma_{r}^{t}\left(P\right)-\gamma_{r}^{*}{\left(P\right)\|}_{2}^{2}$$

where $\delta_{v}^{*}$ is the ground truth (GT) of the affinity field and $\gamma_{r}^{*}$ is the GT of the confidence map; R is the number of confidence maps corresponding to the number of the joints, and V is the number of the two joints connected; W is a binary mask for the GT and set as zero (0) when the pixel P has no GT for the joint to avoid adverse effect on true positive predictions. The loss function f is the sum of δ losses incurred from the first step to $T_{A}$ step and the sum of γ losses incurred from $T_{A}+1$ step to $T_{A}+T_{B}$ step, as shown below:

(5) $$f=\sum_{t=1}^{T_{A}}f_{\delta }^{t}+\sum_{t=T_{A}+1}^{T_{A}+T_{B}}f_{\gamma }^{t}$$

Finally, the model outputs γ that contains the location of the articular point. If γ has multiple similar peak values around the articular point, the non-maximum suppression [23] is used to identify the highest peak value at the articular point.

### 2.2. Object Detection

One of the areas of study in computer vision is object detection technology, which is employed to automatically operate and adjust particular devices. The detection involves classification and localization. In classification, a single object in the image is classified with class probabilities, and localization is a process of determining the location of the object. Object detection methods are largely divided into two-stage detectors and one-stage detectors. The two-stage detector conducts the localization and classification sequentially to obtain the results. In the first stage, the area where an object is likely to present is inferred quickly through the regional proposal. In the second stage, the classification identifies the type of object. The major models include Regions with CNN (R-CNN) [24], Fast R-CNN [25], Faster R-CNN [26], and Mask R-CNN [27]. The two-stage detectors generally have higher accuracy but slow speed.

Unlike the two-stage detector, which performs two processes sequentially, the one-stage detector produces results faster by conducting localization and classification concurrently. The main models include YOLO [19] and Single Shot Multibox Detector (SSD) [28]. YOLO, in particular, significantly enhances the speed of two-stage detectors and can estimate the class probability and the bounding box simultaneously, making it frequently utilized in real-time processing. Furthermore, the training process traverses the full image, learning not just the characteristics of individual objects but also the overall context of the image, resulting in exceptional performance when extending to additional locations. The following steps are taken during the training [19,29,30]: 

After dividing the input image into S×S grid areas, its characteristics are extracted using the convolutional layer, and the prediction tensor is created through the fully connected layer. Each grid cell is represented by the B number of the bounding boxes, each of which has the corresponding confidence score (CS). The bounding box has information about $\left(x,\;y,\;w,\;h,CS\right)$, with $\left(x,\;y\right)$ being the centroid coordinates of the bounding box and $\left(w,\;h\right)$ being its width and height. CS is the probability of the object being within the bounding box and shows whether the class is correctly predicted, as shown below:

(6) $$CS=\mathrm{Pr}\left(Obj\right)\times {\mathrm{IoU}}_{\mathrm{PB}}^{\mathrm{GT}}$$

where $\mathrm{Pr}\left(Obj\right)$ is the probability of the object being within the bounding box; ${\mathrm{IoU}}_{\mathrm{PB}}^{\mathrm{GT}}$, the intersection over the union (IoU), shows the extent to which GT matches the box (PB) determined by the model and corresponds to the overlapping area of the actual value and the predicted value, as shown below:

(7) $${\mathrm{IoU}}_{\mathrm{PB}}^{\mathrm{GT}}=\frac{\mathrm{PB}\;\cap \;\mathrm{GT}}{\mathrm{PB}\;\cup \;\mathrm{GT}}$$

The conditional probability $P_{Class}$ indicating which class multiple objects included in the bounding box belong to and the Class-specific Confidence Score (CCS) indicating the probability that the object is contained within the bounding box area, and it matches with the actual value of the classified object are expressed as follows:

(8) $$P_{Class}=\mathrm{Pr}(Class_{i}|Obj)$$

(9) $$CCS=CS\times P_{Class}=\mathrm{Pr}\left(Class\right)\times {\mathrm{IoU}}_{\mathrm{PB}}^{\mathrm{GT}}$$

As shown above, the bounding box with the highest CCS is finally chosen as the bounding box for the given object among the B number of the bounding boxes predicted.

### 2.3. Object Tracking

Multi-object tracking (MOT) [31,32,33] is a technique for tracking the locations of numerous objects in a video in real time. It first assigns a unique identifier (ID) to each identified object to track its movement by comparing the previous frame and the current frame. Major MOT methods include Simple Online and Realtime Tracking (SORT) [34] and DeepSORT [20]. SORT is a tracking method to analyze only the degree of similarity of the association between objects using only the information of the objects detected in the current frame and the previous frame of the image. However, if the item is obstructed by a barrier during the object tracking, it cannot be identified as the same object indefinitely and thus obtains a new ID that differs from the ID previously assigned. Furthermore, the movement of multiple objects instead of one causes frequent ID-switching, which hinders smooth tracking [20,35].

As an extension of SORT, DeepSORT has object detection, the Kalman filter-based estimation as well as the matching cascade that uses a deep-learning feature Re-ID, and thus addresses the drawbacks of SORT, that is, unstable to occlusion or ID-switching [20]. The Kalman filter is used to update the identified object by estimating its location in the future frame using information from the previous frame. Then, to match the identified object, DeepSORT utilizes the Mahalanobis distance, which gives an object’s location based on the movement effective for short-term prediction, and the cosine distance that uses the object’s appearance for the long-term signaling block followed by the recovery of its identity. We determine the Cost Matrix $D_{CM}$ as the weighted mean of the Mahalanobis distance $D_{MA}$ and the cosine distance $D_{Cos}$ for the calculation of the similarity matrix.

(10) $$D_{CM}=\rho \times D_{MA}+\left(1-\rho \right)\times D_{Cos}$$

where ρ is a hyperparameter used to control the matrix impact; when the camera motion is large, it is set ρ=0, using $D_{Cos}$ only. Then, the IoU matching is performed on the tracks and detections that are not related. The IoU matching process uses three states to obtain information about continuous tracking: matched tracks for objects being tracked continuously, unmatched detections for designating a recently appeared object as the final object, and unmatched tracks for designating an object’s temporary status when the tracked object cannot be found, and the tracking cannot continue.

## 3. Proposed Architecture Design

**Figure 3:**
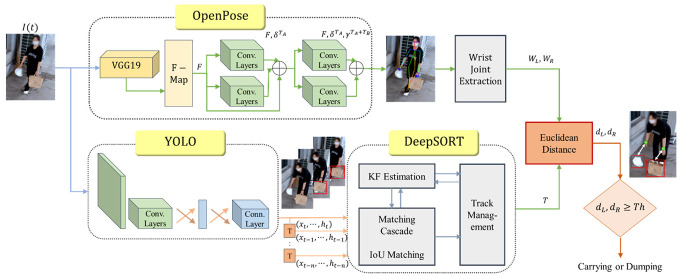
Block diagram of the proposed illegal dumping monitoring system.

In this section, we describe the detailed procedure for designing the proposed monitoring system that detects illegal dumping based on the distance between the potential dumper’s wrist joints found using OpenPose and the garbage bag location obtained through YOLO and DeepSORT. The block diagram in Figure 3 shows the system schematically.

### 3.1. Extraction of the Articular Points of the Wrist Using OpenPose

The articular points of the person’s wrist are retrieved from the video *I*(*t*) of a possible dumper walking into the observation zone while holding the trash. To accomplish this, we input the given image to VGG-19 in OpenPose to generate a feature map, which is then used to generate a confidence map γ for displaying the locations of the joints and an affinity field δ for demonstrating the correlation between the body parts. As we detect illegal dumping based on the point in time when a part of the extracted joints separates from the garbage, in the case of the finger closest to the trash, the next closest wrist joint is selected because the joint coordinates cannot be extracted when the finger is often obscured by other objects. As a result, of the 18 joint coordinates that are retrieved, we only use the elbow and shoulder that are connected to the wrist, and we disregard the remaining 12 coordinates that are beyond the area of interest. The three joints of the shoulder, elbow, and wrist are displayed on the screen in a state where the left arm and the right arm are separated. Then, the joint coordinates of the left wrist $W_{L}$ and the joint coordinates of the right wrist $W_{R}$ are finally estimated.

### 3.2. Tracking the Garbage Bag Using YOLO and DeepSORT

To identify the garbage bag held by the potential dumper, we employ the real-time object detection model YOLO to obtain the bounding box $\left(x,\;y,\;w,h\right)$ of the garbage bag as the identified object. Then, from the bounding box, we extract the top centroid $T\left(t_{1},t_{2}\right)$, which can be expressed as $t_{1}=x+\frac{w}{2},\;t_{2}=y$. Furthermore, to identify illegal dumping in real time, we employ DeepSORT to determine whether the object in the previous frame $I\left(t-1\right)$ and the object in the current frame $I\left(t\right)$ are the same. Here, the Kalman filter, the matching cascade, and the IoU matching [20] are conducted recursively to determine the similarity between each object. Using three states, the matched tracks for the objects being tracked continuously, the unmatched detections for designating a newly discovered object as the final object, and the unmatched tracks for designating a temporary status to the object when the tracked object is not found and the tracking cannot continue, the IoU matching finally defines an ID to the object. Here, the ID contains the types of detected objects and the order $\left(\mathrm{Class}\;\mathrm{name},\;\mathrm{Class}\;\mathrm{number}\right)$ that the objects are detected. This enables the continuous recognition of the same garbage bag even when it is occluded by other obstacles. Moreover, it is possible to suppress the ID switching that may occur due to the movement of multiple garbage bags instead of one garbage bag. Accordingly, even if the detected garbage bag is dumped, it can be made to have the same ID, making a judgment on illegal dumping possible.

### 3.3. Discriminator for the Determination of Illegal Dumping

As described above, to determine the illegality of the garbage bag held by the potential dumper, we compute the Euclidean distance between the wrist joint coordinates $W_{L}\left(w_{L,1},\;w_{L,2}\right)$ and $W_{R}\left(w_{R,1},\;w_{R,2}\right)$ obtained from OpenPose and the top centroid $T\left(t_{1},t_{2}\right)$ of the bounding box obtained from YOLO, as shown below:

(11) $$d_{L}\left(T,W_{L}\right)=\sqrt{{\left(t_{1}-w_{L,1}\right)}^{2}+{\left(t_{2}-w_{L,2}\right)}^{2}}$$

(12) $$d_{R}\left(T,W_{R}\right)=\sqrt{{\left(t_{1}-w_{R,1}\right)}^{2}+{\left(t_{2}-w_{R,2}\right)}^{2}}$$

As the final step, we check if the $d_{L}$ or $d_{R}$ that are calculated per frame exceeds the pre-defined threshold Th to evaluate whether the garbage bag being tracked is dumped illegally. When $d_{L}$ and $d_{R}$ are below the threshold, we set the object ID to 1 to indicate that the potential dumper has the garbage bag. The ID remains 1 while every frame is examined until the point of garbage bag dumping. By contrast, for the garbage bags that are dumped already, $d_{L}$ and $d_{R}$ both surpass the threshold. As a result, we set the object ID to zero (0) to indicate that the garbage bag is not held by the dumper. Thus, immediately after the garbage bag is dumped, that is, when $d_{L}\;\;or\;d_{R}>Th$, a judgment is made that the object is dumped, the ID changes from 1 to 0, and the alarm goes off. Furthermore, as the already dumped garbage bags are detected and set to 0, they are not falsely identified as those being held by the dumper even when the dumper’s wrist gets close to the garbage bag.

## 4. Experimental Results

To assess the performance of the proposed illegal dumping monitoring method, we took into account eight scenarios that were similar to actual instances of illegal dumping, including garbage dumping by one hand, dumping by both hands, garbage dumping without bending the waist, and dumping yet to have occurred with the garbage in the dumper’s hand. We then gathered the data for these cases. Furthermore, to determine the performance against the existing garbage dumping monitoring techniques, we included the approach [7] that learns the dumping postures to decide on illegal dumping and the method, Post+det, that learns the dumping postures as well as the garbage bags. There were a total of eight situations included in the performance test.

### 4.1. Experimental Environment

The proposed illegal garbage dumping monitoring system was implemented by NVIDIA GeForce GTX 1060 Ti and Intel Core i7-8700 CPU. To train YOLOv4 for real-time object detection, we collected illegal dumping films for each situation using a Logitech C920 PRO HD. The dataset includes videos of the simulation of actual illegal dumping scenes, with 30 videos of about 10 s for each scenario.

**Figure 4:**
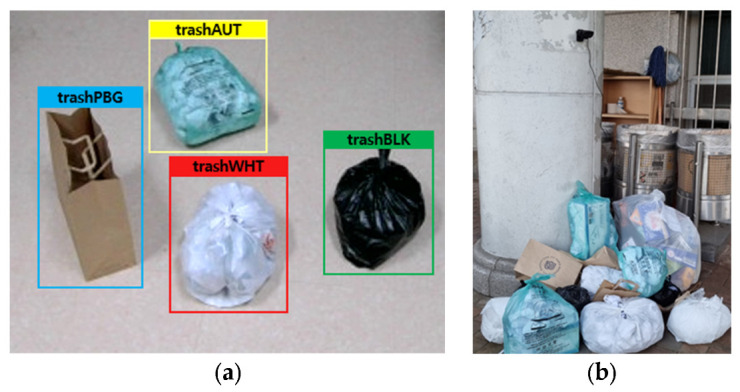
The environment for the collection of illegal dumping data and scenario-based evaluation; (**a**) standard and non-standard garbage bags used in the training and the evaluation; (**b**) data collection environment.

Commonly dumped garbage includes black plastic bags, white plastic bags, and paper bags containing general garbage, as well as volume-based bags that are recommended to be used. We selected four types of bags that are dumped the most, as shown in Figure 4a, to simulate actual dumping scenes under the environment in Figure 4b. We labeled the black plastic bag trashBLK, the white plastic bag trashWHT, the paper bag trashPBG, and the standard bag trashAUT. For the YOLOv4 training, we utilized a total of 12,891 images, with the image size set to 608×608, the batch size to 8, and the maximum number of batch learning to 15,000. There may be several items in a single photograph. There are 13,186, 16,147, 15,611 and 11,711 trashBLK, trashWHT, trashPBG, and trashAUT in all of the photos, respectively.

### 4.2. Evaluation of Object Detection Performance

We used the average precision (AP) as a performance indicator for assessing the performance of the object detection model YOLOv4, which is trained on the different types of collected garbage bags. To denote the model’s performance as a single numerical value, we utilized the precision-recall curve and the accuracy to evaluate the confidence of the object identified by the model. Precision is the rate of the correctly detected objects among the detected objects, recall is the rate of the detected objects among all the objects that should be detected, and accuracy is the rate of the correctly detected objects among all the objects, as demonstrated below [6]:

(13) $$\mathrm{Precision}=\frac{\mathrm{TP}}{\mathrm{TP}+\mathrm{FP}}$$

(14) $$\mathrm{Recall}=\frac{\mathrm{TP}}{\mathrm{TP}+\mathrm{FN}}$$

(15) $$\mathrm{Accuracy}=\frac{\mathrm{TP}+\mathrm{TN}}{\mathrm{TP}+\mathrm{FP}+\mathrm{FN}+\mathrm{TN}}$$

where the True Positive (TP) means the object that should be identified is correctly detected, the False Positive (FP) means the object that should not be detected is wrongly detected, the False Negative (FN) means the object that should be detected is not detected, and the True Negative (TN) means the object that should not be detected is not detected. Seven hundred and ninety-eight images were used to determine object detection, and the results are shown in Table 1.

As illustrated in the table, when the IoU is 0.5, the detection performance indicator, AP, for each class is mostly above 99%, while the average indicator, meanAP (mAP), for all classes is 99.38%, indicating that the model can classify all four objects with high accuracy. However, trashBLK indicates a lower precision than the other types of garbage bags due to the occasional false recognition of a person’s black hair or shoes.

### 4.3. Evaluation of the Illegal Dumping Monitoring Performance

**Figure 5:**
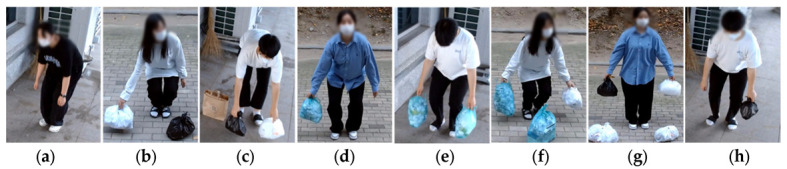
Eight types of scenarios (S1~S8) for the performance evaluation of the illegal dumping monitoring system; (**a**) S1—a bending posture with no garbage bag; (**b**) S2—a dumping scenario with the non-standard bag in one hand; (**c**) S3—a dumping position with the non-standard garbage bags on both hands; (**d**) S4—a dumping posture with the legal standard garbage bag in hand; (**e**) S5—a dumping scenario with legal standard garbage bags in both hands; (**f**) S6—a dumping posture with the standard bag in one hand and the non-standard bag in the other hand; (**g**) S7—a dumping posture without bending the waist with the non-standard bags in both hands; and (**h**) S8—a bending position without dumping with the non-standard bag.

The data gathered for the evaluation has a total of four types of garbage bags previously described. As shown in Figure 5, we developed eight different dumping scenarios, S1 through S8, which are comparable to real garbage dumps.

The proposed AIDM determines illegal dumping based on the distance ($d_{L},\;d_{R}$) between the wrist joints of a dumper and the detected object, not the dumping posture. To achieve this, we established a threshold (Th) to 90 cm, taking into account the installation angle and the distance between the camera and the visible object. To verify the utility of the proposed method, we performed a comparison against the existing monitoring techniques: the technique [7] that determines whether illegal dumping has occurred solely based on a dumping posture with the body bent forward, and the technique, Post+det, that monitors illicit dumping through the detection of garbage and dumping postures. The test results are reported in Table 2 in terms of the reliability of the determination of illegality at the site of dumping using the scenarios S1 to S8.

As can be seen from the comparison, [7] recorded a lower accuracy in the scenarios S1, S4, S5, and S7 because it determines whether dumping is legal by learning the shapes of the dumpers rather than the garbage bags, in contrast to the Post+det and the ADIM, which can identify the standard bags that can be legally dumped. Furthermore, the Post+det appears to demonstrate a higher detection performance overall than [7]. However, it occasionally failed to detect suspicious dumping actions, leading to lower accuracy in scenarios S2, S3, and S6. Particularly for S7, it failed to detect anything since the garbage dumping occurred without bending the body. In contrast, the proposed model demonstrated at least 93% accuracy in identifying illegal dumping in all the scenarios, demonstrating that it is a stable illegal dumping monitoring system. On the whole, the average accuracy of [7], the Post+det, and the AIDM for detecting illegal dumping are 0.43, 0.63, and 0.97, respectively. Therefore, it can be said that the proposed AIDM has a more robust and improved detection performance than the existing method.

**Figure 6:**
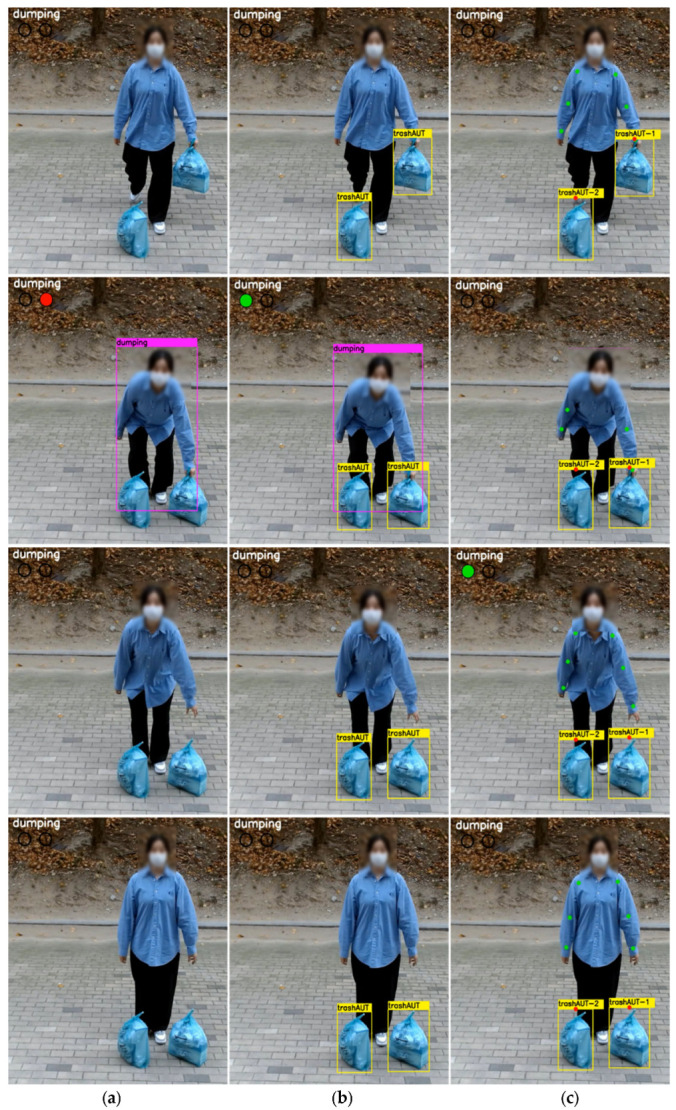
Illustrations of the outcomes from the monitoring models for the disposal of the legal standard garbage bag in one hand; (**a**) [7], (**b**) Post+det, and (**c**) Proposed AIDM.

Figure 6 shows the test results for scenario S4, where a legal volume-based waste bag is thrown on one hand. From top to bottom, the results are taken from each time point of T/4-, T/2-, 3T/4-, and T-seconds. At T/4~T/2 s, the dumper is shown walking with the garbage in hand to the designated dumping site. In Figure 6a, there is no change since the dumper has to bend his body for the dumping to be detected as such. In Figure 6b, the system found the legal standard bag trashAUT, and in Figure 6c, it concurrently located the person’s joints and detected trashAUT. The dumper dropped the trash bag at the 3T/4-s point. [7] detected the dumping posture only and not the type of garbage bag, identifying it as illegal and indicating the red alarm. On the other hand, the Post+det and the AIDM can differentiate the standard bag, showing the green alarm after detecting the dumping action and deeming it legal. The T-second mark is the moment right before the dumper departs the site after dumping the garbage. The alarm was no longer displayed in [7] and the Post+det for garbage dumping as the dumper stopped bending their body, whereas the AIDM kept the green alarm as the garbage bag discarded by the dumper had a unique ID.

**Figure 7:**
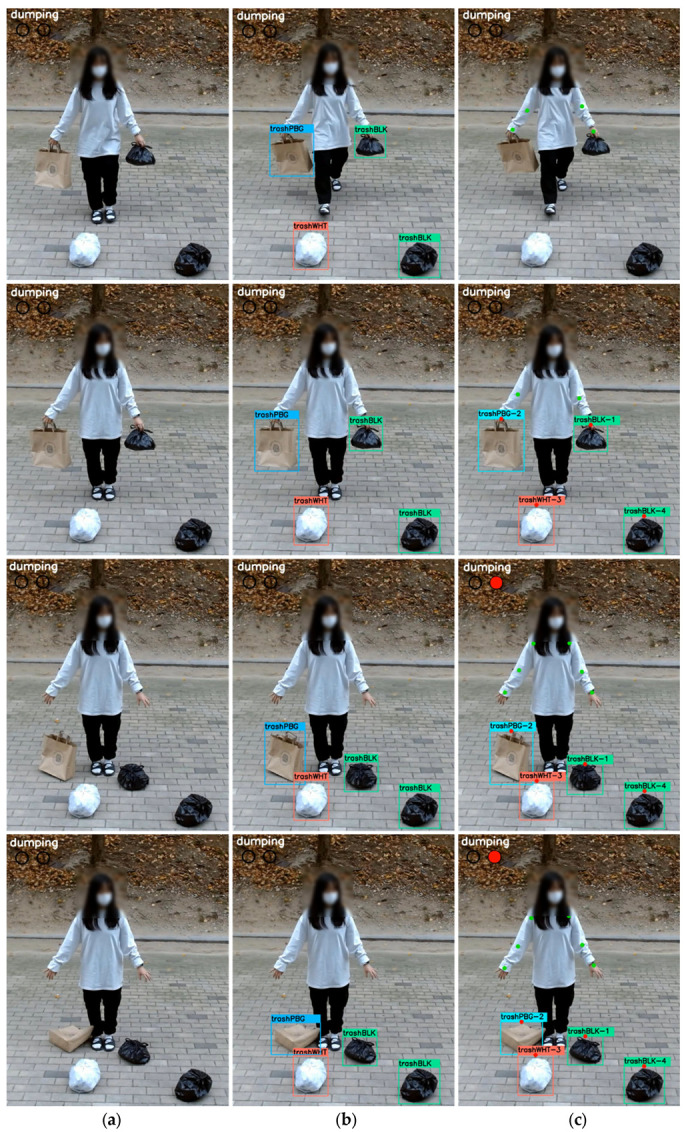
Examples of the findings from the monitoring models for rubbish disposal without body bending; (**a**) [7], (**b**) Post+det, and (**c**) Proposed AIDM.

Figure 7 additionally demonstrates the test results for scenario S7, where the dumper dumps the non-standard garbage bags without bending their body. Similar to the above instances, at T/4~T/2 s, [7] did not identify anything, while the Post+det detected three types of garbage bags, trashBLK, trashWHT, and trashPBG. The AIDM found the person’s articular points and, like the Post+det, detected all three types of garbage bags. At the 3T/4-second mark, in which the garbage is dumped, [7] the Post+det failed to detect a dumping action as the dumper did not bend his body. On the other hand, the AIDM identified the non-standard garbage bag and determined that the distance from the wrist to the bag was above the threshold, thus deeming it unlawful and showing the red alarm.

## 5. Conclusions

The government of the Republic of Korea has implemented a volume-based waste disposal system that can change the disposal features to efficiently handle massive amounts of waste. However, illegal dumping often occurs as people dump garbage in disposable black plastic bags or white plastic bags used for food deliveries. Recently, methods have been implemented in areas where illegal garbage dumping occurs to control such behavior by installing closed-circuit television (CCTV) and the transmission of audio warnings using human body detection sensors. Nevertheless, the effect is limited. As a result, numerous actions are necessary since unlawful dumping is constantly growing in the absence of strict prosecution. Therefore, this study suggested a deep neural network-based illegal dumping monitoring technique that can determine the distance between the dumper’s wrist and the garbage bag. The proposed technique retrieves the articular points of a dumper using OpenPose and identifies the type of garbage bag through the object detection model YOLO. Furthermore, to reduce false detection of illegal dumping, we introduced a method of tracking the IDs issued to the waste bags using the MOT model. The test results demonstrate that our approach of determining illegal dumping based on the distance of the actual dumper’s wrist to the garbage bag has better performance than other recently published methods based on behavior recognition or dumping zone designation. We expect the proposed method to be widely utilized in the future.

## Figures and Tables

**Table 1:** Performance of identifying the four types of garbage bags using YOLOv4.

Class ID	Object	AP	TP	FP
0	trashBLK	99.77%	685	36
1	trashWHT	99.53%	706	7
2	trashPBG	98.96%	823	7
3	trashAUT	99.24%	712	8

**Table 2:** Performance comparison of dumping monitoring models per scenario.

Test Scenario	Dumping Monitoring Model														
	[7]					Post+det					Proposed AIDM				
	TP	TN	FP	FN	Acc.	TP	TN	FP	FN	Acc.	TP	TN	FP	FN	Acc.
S1	0	1	29	0	0.03	0	30	0	0	1.00	0	30	0	0	1.00
S2	25	0	0	5	0.83	17	0	0	13	0.57	28	0	0	2	0.93
S3	28	0	0	2	0.93	7	0	0	23	0.23	30	0	0	0	1.00
S4	0	7	23	0	0.23	0	28	2	0	0.93	0	30	0	0	1.00
S5	0	1	29	0	0.03	0	25	5	0	0.83	0	30	0	0	1.00
S6	28	0	0	2	0.93	17	0	0	13	0.57	29	0	0	1	0.97
S7	2	0	0	28	0.07	0	0	0	30	0.00	29	0	0	1	0.97
S8	0	12	18	0	0.40	0	28	2	0	0.93	0	28	2	0	0.93
Average Acc.	0.43					0.63					0.97				

## Data Availability

The data presented in this study are available on request from the corresponding author.

## References

[1] Park J. (2000). An Evaluation of Volume-Based Waste Collection Fee System. Master’s Thesis.

[2] Kim D. (2006). A Study on Improvement Plan for Trash Specific Duty. Master’s Thesis.

[3] Kim J. (2003). A Study on the Estimation for Improvement of the Volume based Waste Fee System. Master’s Thesis.

[4] Seoul Information Communication Plaza Performance of Cracking Down on Illegal Dumping of Garbage. https://opengov.seoul.go.kr/.

[5] Mu J. (2016). A Study on Improving Household Waste Collection Systems. Master’s Thesis.

[6] Min H., Lee H. (2021). Garbage Dumping Detection System using Articular point Deep Learning. J. Korea Multimed. Soc..

[7] Bae C., Kim H., Yeo J., Jeong J., Yun T. Development of Monitoring System for Detecting Illegal Dumping Using Deep Learning. Proceedings of the Korean Society of Computer Information.

[8] Jeong J., Kwon S., Kim Y., Hong S., Kim Y. Development of Illegal Dumping System using Image Processing. Proceedings of the Korean Institute of Information Scientists and Engineers.

[9] Kim J., Kim H., Kim P., Lee Y. The Design of Intelligent System for Statistically Determining Illegal Garbage Dumping through Trajectory Analysis. Proceedings of the Korean Institute of Information Scientists and Engineers.

[10] Ramanan D., Forsyth D., Zisserman A. Tracking people and recognizing their activities. Proceedings of the IEEE Conference on Computer Vision and Pattern Recognition.

[11] Yang Y., Ramanan D. (2013). Articulated Human Detection with Flexible Mixtures of Parts. IEEE Trans. Pattern Anal. Mach. Intell..

[12] Lan X., Huttenlocher D. Beyond trees: Common-factor models for 2D human pose recovery. Proceedings of the IEEE International Conference on Computer Vision.

[13] Dantone M., Gall J., Leistner C., Van Gool L. Human Pose Estimation Using Body Parts Dependent Joint Regressors. Proceedings of the IEEE Conference on Computer Vision and Pattern Recognition.

[14] Tompson J., Goroshin R., Jain A., LeCun Y., Bregler C. Efficient Object Localization using Convolutional Networks. Proceedings of the IEEE Conference on Computer Vision and Pattern Recognition.

[15] Chen Y., Shen C., Wei X.-S., Liu L., Yang J. Adversarial PoseNet: A Structure-Aware Convolutional Network for Human Pose Estimation. Proceedings of the IEEE International Conference on Computer Vision.

[16] Sun M., Savarese M. Articulated part-based model for joint object detection and pose estimation. Proceedings of the IEEE International Conference on Computer Vision.

[17] Fang H.-S., Xie S., Tai Y.-W., Lu C. RMPE: Regional Multi-person Pose Estimation. Proceedings of the IEEE International Conference on Computer Vision.

[18] Cao Z., Hidalgo G., Simon T., Wei S.E., Sheikh Y. (2021). OpenPose: Realtime multi-person 2D pose estimation using part affinity fields. IEEE Trans. Pattern Anal. Mach. Intell..

[19] Redmon J., Divvala S., Girshick R., Farhadi A. You only look once: Unified, real-time object detection. Proceedings of the IEEE Conference on Computer Vision and Pattern Recognition.

[20] Wojke N., Bewley A., Paulus D. Simple Online Realtime Tracking with a Deep Association Metric. Proceedings of the IEEE Conference on Image Processing.

[21] Badave H., Kuber M. Evaluation of Person Recognition Accuracy based on OpenPose Parameters. Proceedings of the International Conference on Intelligent Computing and Control Systems.

[22] Simonyan K., Zisserman A. (2015). Very Deep Convolutional Networks for Large-Scale Image Recognition. arXiv.

[23] Hosang J., Benenson R., Schiele B. Learning Non-maximum Suppression. Proceedings of the IEEE Conference on Computer Vision and Pattern Recognition.

[24] Girshick R., Donahue J., Darrell T., Malik J. Rich Feature Hierarchies for Accurate Object Detection and Semantic Segmentation. Proceedings of the IEEE Conference on Computer Vision and Pattern Recognition.

[25] Girshick R. Fast R-CNN. Proceedings of the IEEE International Conference on Computer Vision.

[26] Ren S., He K., Girshick R., Sun J. (2015). Faster R-CNN: Towards real-time object detection with region proposal networks. IEEE Trans. Pattern Anal. Mach. Intell..

[27] He K., Georgia G., Dollar P., Girshick R. Mask R-CNN. Proceedings of the IEEE International Conference on Computer Vision.

[28] Liu W., Anguelov D., Erhan D., Szegedy C., Reed S., Fu C.Y., Berg A.C. SSD: Single shot multibox detector. Proceedings of the European Conference on Computer Vision.

[29] Bochkovskiy A., Wang C.Y., Liao H.Y.M. (2020). Yolov4: Optimal speed and accuracy of object detection. arXiv.

[30] Akyol G., Kantarcı A., Çelik A., Cihan Ak A. Deep Learning Based, Real-Time Object Detection for Autonomous Driving. Proceedings of the IEEE Conference on Signal Processing and Communications Applications.

[31] Teknomo K., Takeyama Y., Inaura H. Frame-based tracing of multiple objects. Proceedings of the IEEE Workshop on Multi-Object Tracking.

[32] Luo W., Xing J., Milan A., Zhang X., Liu W., Kim T.K. (2017). Multiple Object Tracking: A Literature Review. arXiv.

[33] Wang Z., Zheng L., Liu Y., Li Y., Wang S. Towards Real-Time Multi-Object Tracking. Proceedings of the European Conference on Computer Vision.

[34] Bewley A., Ge Z., Ott L., Ramos F., Upcroft B. Simple Online and Realtime Tracking. Proceedings of the IEEE International Conference on Image Processing.

[35] Pereira R., Carvalho G., Garrote L., Nunes U.J. (2022). Sort and Deep-SORT Based Multi-Object Tracking for Mobile Robotics: Evaluation with New Data Association Metrics. Appl. Sci..

